# Comparison of Predicted Probabilities of Proportional Hazards Regression and Linear Discriminant Analysis Methods Using a Colorectal Cancer Molecular Biomarker Database

**Published:** 2007-03-02

**Authors:** Sreelatha Meleth, Chakrapani Chatla, Venkat R. Katkoori, Billie Anderson, James M. Hardin, Nirag C. Jhala, Al Bartolucci, William E. Grizzle, Upender Manne

**Affiliations:** 1 Biomedical Statistics, Department of Medicine;; 2 Department of Pathology, University of Alabama at Birmingham, Birmingham, Alabama;; 3 Department of Information Systems, Statistics, and Management, University of Alabama at Tuscaloosa, Alabama;; 4 Department of Biostatistics, University of Alabama at Birmingham, AL

**Keywords:** predictive models, linear discriminant analysis, proportional hazards regression, colorectal cancer, survival

## Abstract

**Background:**

Although a majority of studies in cancer biomarker discovery claim to use proportional hazards regression (PHREG) to the study the ability of a biomarker to predict survival, few studies use the predicted probabilities obtained from the model to test the quality of the model. In this paper, we compared the quality of predictions by a PHREG model to that of a linear discriminant analysis (LDA) in both *training* and *test set* settings.

**Methods:**

The PHREG and LDA models were built on a 491 colorectal cancer (CRC) patient dataset comprised of demographic and clinicopathologic variables, and phenotypic expression of p53 and Bcl-2. Two variable selection methods, *stepwise discriminant analysis* and the *backward selection*, were used to identify the final models. The endpoint of prediction in these models was five-year post-surgery survival. We also used linear regression model to examine the effect of bin size in the *training set* on the accuracy of prediction in the *test set*.

**Results:**

The two variable selection techniques resulted in different models when stage was included in the list of variables available for selection. However, the proportion of survivors and non-survivors correctly identified was identical in both of these models. When stage was excluded from the variable list, the error rate for the LDA model was 42% as compared to an error rate of 34% for the PHREG model.

**Conclusions:**

This study suggests that a PHREG model can perform as well or better than a traditional classifier such as LDA to classify patients into prognostic classes. Also, this study suggests that in the absence of the tumor stage as a variable, Bcl-2 expression is a strong prognostic molecular marker of CRC.

## Introduction

One of the consequences of the arrival of high though-put technological platforms in biomarker discovery research and resultant generation of high-dimensional datasets has been a re-focusing of analysts’ attentions on the quality of predictions from models and on measures of predictive accuracy. Studies that develop predictive models now routinely address the issues related to the validation of findings by analyzing the *‘training’* and ‘*test’* sets or utilize cross validation approaches like “*leave one out cross validation*”(1).

Classification is a process which assigns objects to a category, and is routinely used in many scientific studies. Fisher and his contemporaries were the pioneers in the development of classifiers such as the *Linear discriminant analysis* (LDA) and *cluster analysis* (2). The LDA uses the distance of an individual’s mean value from the estimated mean of a group in order to classify that individual into a group. The closer the individual’s result is to a group’s mean value the greater is the probability of the individual belonging to that group. These classifiers can easily be implemented in commonly used statistical software tools, including *SAS* and *S-Plus*.

PHREG models are the multivariable models that are most commonly used in studies that evaluate molecular markers to predict cancer prognosis, or compare efficacy of cancer therapies. This is because the outcome of interest in most cancer studies is time to an ‘*event’* such as death, recurrence, or metastasis. PHREG is a semi-parametric model, which assesses the effect of various independent variables on the time to an event of interest. One of the problems encountered in the accumulation of data on individuals over a time period is that patients are often either lost to follow up or with unknown outcomes. The contribution of these individuals to the model, as long as there is a credible data on them, is considered to be an advantage of the PHREG model which allows for ‘*censoring*.’ The hazard ratios estimated by these models are also widely used, and easily interpreted both by researchers and clinicians.

The objective of this study was to compare the predictions of a PHREG model of 5-year cancer-specific mortality to that of the LDA. Since the PHREG model probability estimates are adjusted for censored individuals, we hypothesized that a classifier based on predicted probabilities estimated by PHREG would probably perform better than an LDA. Two separate variable selection techniques (stepwise discriminant analysis and backward selection) were used to identify final models in both cases. We also examined the possibility of identifying variables which can be used to predict the clinical outcome or aggressive behavior of CRC by clinicians well before the pathologic staging information is available. For this, we tested the molecular phenotypic markers, nuclear accumulation of p53 (p53^nac^) and expression of Bcl-2, in models without the tumor stage variable to assess their prognostic value and compared to the value of the tumor stage in predicting survival of CRC patients.

## Patients and Methods

### Patients

The institutional review boards of the University of Alabama at Birmingham (UAB) and its affiliated Birmingham Veterans Affairs (VA) Hospital approved this study. We identified a total of 491 patients from the UAB and VA Hospital tumor registries who had undergone surgical resection only for ‘*first primary*’ CRC from 1981 through 1993. None of these patients received any pre- or post-surgery chemo or radiation therapies for various reasons. We obtained the medical records including surgical pathology reports of these patients which were reviewed by two of the authors (CC and UM) to ascertain the key information. The patient cohort from both hospitals was under the care of a uniform group of physicians.

## Pathological features

In our study, two pathologists (NCJ and WEG) reviewed hematoxylin and eosin stained slides of all cases for the degree of histologic differentiation and graded as well, moderate, poor or undifferentiated. Subsequently, we pooled well and moderately differentiated tumors into a low grade group and poor and undifferentiated tumors into a high grade group (3). The pathologic staging was performed according to the criteria of the American Joint Commission on Cancer (4) using the information extracted from the pathology reports. In our study, we pooled Stages I and II and Stage III and Stage IV into node negative and node positive categories, respectively. The International Classification of Diseases for Oncology (ICD-O) codes were used to specify anatomic location of the tumor (5). The anatomic sub-sites were grouped into the proximal colon, the distal colon and the rectum.

## Follow-up

Patients were followed by the UAB and VA tumor registries until their death or the date of the last documented contact within the study time frame. The tumor registries ascertain outcome (mortality) information directly from patients (or living relatives) and from the physicians of the patients through telephone and mail contacts. This information is further validated against State Death Lists. The tumor registries update follow-up information every six months and follow-up of our cohort ended in September 2006. There was only one individual who was censored due to loss to follow up in the test data set. All the other censored individuals are censored because they were alive for more than five years at the last follow up. This quality of follow-up for all cause mortality allows us to estimate the sensitivity and specificity of the two models for 5 year all cause mortality with a great degree of accuracy.

### Immunohistochemistry

Formalin-fixed, paraffin-embedded archival tissues of CRCs were collected from the surgical pathology division of UAB and VA Hospitals. Immunohistochemical staining and immunostaining evaluation of nuclear accumulation of p53 and Bcl-2 were described earlier (6–10). The molecular marker expression was dichotomized into high expressers and low expressers based on the cut-off values discussed below. For Bcl-2 expression, based on our prior studies (9, 11), 0.5 ISS was chosen as the cut-off value. We considered only tumor cells with distinct nuclear immunostaining for p53 as positive and considered the tumor positive only if ≥10% positivity of all malignant cells in a tissue section as described earlier (7).

### Statistical analyses

The outcome variable LDA model was a binary variable indicating cancer-specific survival (or not) five years post-surgery for CRC. Time to Cancer-specific death was the outcome for the PHREG model. The censoring variable for the PHREG model was a dichotomous variable by the PHREG model that identified individuals who had died as a result of CRC in the five years after surgery. Individuals who died within five years due to other causes or those who lived more than five years were considered censored. The training set had data on 234 non-Hispanic Caucasians (80% of initial 292), and 159 (80% of 199) African-Americans who were randomly selected from 491 patients. The remaining individuals formed the test set. Forty-one percent (162) of individuals in the training data set had the event of interests and 59 % were censored. The proportion of censored individuals in the test data set was 39%. The variables available for inclusion in the model were, age (< 65 vs. ≥ 65 years), race (non-Hispanic Caucasians vs. African-Americans), tumor stage (I & II vs. III & IV or node negative vs. node positive), tumor differentiation (low grade vs. high grade), location of tumor (proximal colon, distal colon and rectum), and p53nac and Bcl-2 expression. All variables were dichotomized for an easier interpretation of the hazard ratios. Appropriate cut-points for both the molecular markers as well as continuous variables such as age based on this data have been identified in previous published studies based on this data (6–10). We first used a bootstrapped stepwise discriminant analysis as a variable selection technique. This technique is loosely based on the pre-validation technique described by Tibshirani and Efron (12). We have previously described this method in our analysis of the data generated by Surface Enhanced Laser Absorption/Desorption Ionization—Time of Flight (SELDI-TOF) (13). In this, 75% of the training set was randomly selected and subjected to a stepwise discriminant analysis procedure to identify those variables that could best separate the two groups (those who did or did not survive five years post-surgery). This list of variables was stored and the step was iterated 1000 times. Variables that occurred in at least 500 of the 1000-stored lists were selected for inclusion in the final LDA models. The process was first run by including the tumor stage in the list of variables in the stepwise discriminant procedure, and then again after omitting the tumor stage from the list of variables available for selection. Since Stepwise Discriminant Analysis does not account for censoring, this iterative re-sampling technique provides a mechanism for censored and uncensored individuals to contribute equally in the selection of a final model. The backward selection technique available in SAS (V9.1.3)®’s Proc PHREG was used as a second variable selection technique. Five year survival probabilities were estimated using both LDA and PHREG. In SAS (V9.1.3)® Proc Discrim was used to classify the individuals using the LDA. With three dichotomous variables in the model, an individual will have one of eight possible combinations of the variables. A categorical variable that identified these eight combinations was created and each individual was assigned to one of the categories. PHREG estimated a predicted probability for survival for each of the eight categories based on the model built on the training set. A similar procedure was followed for the two variable models which only had four possible combinations of the two dichotomous variables. In both, the LDA and the PHREG, if an individual’s predicted probability of survival beyond five years was ≤ 0.5, that individual was considered a non-survivor. The proportion of survivors correctly identified (specificity), the proportion of non-survivors correctly identified (sensitivity), and the average error rate of the model (the average proportion of incorrectly identified survivors and non-survivors) were used to compare the quality of the models. We also examined the effect of the proportion of individuals in a bin in the training set to the accuracy of the prediction in that category in the test set utilizing the linear regression method. A bin is a particular combination of the variables in the model; e.g. in model 1 (Table 1) all individuals with a tumor that is a stage 1 or 2, negative for Bcl-2, and well differentiated belong to one bin (row 1, Table 1). Thus each row of Table 1 defines a bin for the particular model. The proportion of correct predictions in both LDA and PHREG were very close in most models in most bins. We have only demonstrated the effect of bin size on prediction accuracy based on the PHREG model.

## Results

This variable selection procedure using an iterative stepwise discriminant analysis resulted in final models that had two predictors (tumor stage and Bcl-2 expression) when tumor stage was included in the initial list and two predictors (Bcl-2, tumor differentiation) when stage was excluded. When the backward selection procedure in PHREG was used as a variable selection technique, the two final models were, a three variable model (tumor stage, Bcl-2, and tumor differentiation) when tumor stage was included in the variable list, and a two variable model (Bcl-2 and tumor differentiation) when stage was excluded. Table 2 displays the results of the PHREG models fit to the training data. Bcl-2 was a significant predictor of five-year survival in all the models in this study. It was identified as a significant predictor in models that included tumor stage as well as those that excluded it. It was identified as a significant variable by both types of variable selection techniques. When the tumor stage was included in the list of possible predictors, as expected both variable selection techniques selected tumor grade as a significant predictor of five-year survival. The PHREG backward selection procedure identified tumor differentiation in addition to tumor stage and Bcl-2 expression as a significant predictor of five-year survival. Table 1 displays the probability of survival beyond five years as predicted by the PHREG model, and the LDA model, for an individual with a given combination of variables. This table is divided into three sections for the three final models that resulted from the two variable selection procedures used. The table also displays the survivors and non-survivors correctly identified by the different models.

**Figure 1.**
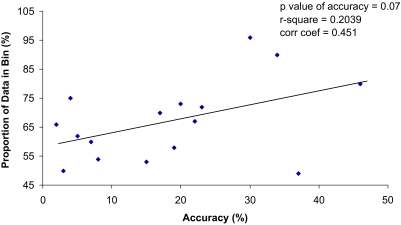
**Accuracy of Prediction in Test Set versus Bin Size in Training Set.** The X-axis represents proportion of true deaths and true survivors accurately predicted in each bin in the *test data set*. The y-axis represents the proportion of individuals in each bin in the training data set. A bin is a particular combination of the variables in the model; e.g. All individuals with tumor stage I or II, negative for Bcl-2 expression, and with well differentiated tumors belong to one bin.

The regression of the proportion of correct predictions in each bin in the test set versus the proportion of individuals in the bin in the training set is shown in Figure 1. The correlation coefficient is 0.451, with a *p*-value of 0.07. The Beta coefficient for the proportion of individuals in a particular bin in the training set is 0.4632, suggesting that unit increase in the proportion of the individuals in a bin in the training set will result in an increase in the accuracy of prediction in the test set.

## Discussion

The current study has demonstrated that a PHREG model built on a training set can be utilized to classify individuals in a test set. This study used both the traditional stepwise procedures generally used for PHREG models to select the variables to be entered into the model as well as an iterative technique that used stepwise discriminant analysis as variable selection techniques. At this point of time, this method, using a PHREG model to estimate predictability, may be limited to models built on dichotomized variables; however, our studies to implement this approach to continuous variables are currently in progress.

In our current study, we have considered the predictions of Cox’s PHREG and LDA multivariable models. A proper comparison of these models requires the comparison of predictions based on all the variables in the model. The accuracy of the predictions in the *test set* in this study was assessed by comparing the predicted probability of death to the observed truth. This is a more accurate test for the predictive quality of a model than the visual determination that considers whether the predicted curves are farther apart or closer together. Therefore, the current study has focused on comparing these two techniques that are commonly accessible to the data analysts in routinely used statistical software applications, including SAS and S-Plus, for the analysis of biomedical databases.

In the models that included TNM stage (depth of tumor, pT; regional lymph node metastasis, pN; and distant metastasis, M), the predicted probabilities from these two models, the PHREG and LDA, were very similar to each other. In fact the classification of individuals based on these estimated probabilities was identical, i.e. PHREG and LDA identified the same individuals as survivors and non-survivors. This finding was contrary to our expectations; because one would expect that the PHREG with its ability to allow for censoring in the training dataset would perform better in predicting the five-year survival than the LDA which has no mechanism to adjust for censoring. However, in the models that excluded tumor stage, PHREG model did perform better than the LDA in predicting non-survivors. The PHREG model identified individuals that were likely to die in five years with a much better accuracy than the LDA model. One explanation for this observation is that the strength of the prognostic value of TNM stage overrides any benefits that accrue from the censoring mechanism in PHREG. However, in the absence of tumor stage, as expected, the predicted probabilities from PHREG allowed better identification of survivors and non-survivors. This is an important finding specifically for prognostic variables such as molecular marker, gene expression or protein expression data. Most of these variables are expected to have modest individual prognostic effects. In models that seek to predict survival/ recurrence using such variables, this study suggests that the predicted probabilities from PHREG are better at classifying the prognosis of an individual with yet unknown consequences.

Additional significant finding of this study is that Bcl-2 expression as a strong predictor of five-year survival in CRC patients. Specifically, these findings have far reaching clinical implications in the treatment of CRC particularly in predicting the outcome of patients who undergo the excisional biopsy as therapy. Because, information on all components of TNM staging can not be obtained from histologic assess of biopsy specimens; thus, these findings might be useful in assessing the aggressiveness of the tumor at the time of biopsy.

Currently, treatment decisions are made based on clinical and pathologic stage of CRC; however, these features may not be the best indicators of prognosis, since groups of patients with tumors of identical stage have different treatment responses and outcomes. In fact, the TNM stage is probably more of a reflection on how long a tumor has been developing rather than the biological features which lead to aggressiveness of tumors (14). Therefore, more recent studies have focused on developing predictive/prognostic molecular markers in CRC which mirror the biology of lesions. However, no marker used to predict the responses of patients to specific therapies (predictive) or assess their survival (prognostic) has been universally accepted to be clinically useful due to contradictory results. These controversies were due to variations in techniques used to assess the status of molecular markers, treatment modalities, underpowered study populations, admixture of different proportions of tumors with different pathologic stages, etc. Therefore, to identify markers which predict the clinical outcomes with greater accuracy, our group has developed large CRC databases and have previously demonstrated the potential value of p53^nac^, Bcl-2, MUC1 and p27^kip-1^ expression in predicting the survival of subgroups of patients with CRC (7, 9, 15, 16).

In the initial training set, p53^nac^ was not identified as a potential prognostic molecular marker. This is most likely because of p53^nac^ has been identified as a prognostic molecular maker only for a subset of CRCs, specifically for proximal colonic adenocarcinomas from non-Hispanic Caucasian patients (17). Similarly, the hazard ratio for low Bcl-2 might have been higher if specified subsets of tumors had been evaluated. Specifically, Bcl-2 expression does not seem to be as strong a prognostic biomarker in African-American patients (11).

For the successful implementation of this method of predictability, one should consider the standard issues of the predictive modeling processes. Since the number of variables in the model increases, the number of possible combinations increases, and the number of individuals with each combination will decrease. This increases the possibility that the predicted probabilities are less stable (have a larger variance), which in turn could reduce the predictive quality of a model. As the number of variables increase the sample size in the training set needs to be sufficiently larger in order to ensure the reliability of this model to predict probability of patient survival. We have tried to demonstrate this fact by using the data available to us in this study. Given the eight bins in the 3-variable model and 4 bins each in the two two-variable models, we had only 16 data points to fit this model. In order to see a statistically significant correlation with 80% power the correlation between the two variables in the regression would have to have been at least 0.80. Figure 1 shows that that the accuracy of predictions in the test set is a function of the bin size in the training. The *p*-value (*p* = 0.07) of the correlation between bin size and accuracy in this small sample example, illustrates the importance not only of having a sufficient proportion of individuals in a each bin in the training set.

The current study has built models on these CRC databases to test the ability of the PHREG and LDA models in predicting probabilities. The findings of this study have demonstrated the usefulness of PHREG models in building classifiers. The accuracy of the classification by PHREG was better than the LDA classification in the absence of the TNM stage variable. This study suggests that a classifier with modest, but important prognostic, effects the ability of the PHREG model to use information from censored individuals improves the predictive quality of the model. Based on this study, it is reasonable to conclude that appropriate model variable selection has a significant effect on the predictive ability of the model. Additionally, these studies have identified clinically important findings that patient age as a significant prognostic indicator even in the models which included tumor stage as a variable. These findings also suggest that it might be possible to use expression of Bcl-2 as a molecular marker to predict the patient prognosis when the information on tumor stage (TNM) is not available; specifically, useful in assessing the aggressiveness of the tumor at the time of biopsy.

## Figures and Tables

**Table 1.** Predicted Probabilities for the Combinations of Variables in PHREG and LDA.

**Model 1: *Predictors - Tumor Stage, Bcl-2 & Tumor grade; Variable Selection Technique—PHREG Backward Selection***		
***Combination of Variables**^a^ (Tumor stage^b^, tumor grade^c^ & Bcl-2 expression^d^)*	**PHREG^e^**	**LDA^e^**
I & II + Low + Bcl-2 negative	0.69	0.72
III & IV + Low + Bcl-2 negative	0.27	0.35
I & II + High + Bcl-2 negative	0.55	0.38
I & II + Low + Bcl-2 positive	0.78	0.86
III & IV + High + Bcl-2 negative	0.11	0.07
I & II + High + Bcl-2 positive	0.67	0.53
III & IV + Low + Bcl-2 positive	0.41	0.47
III & IV + High + Bcl-2 positive	0.23	0.08
*% deaths correctly predicted*	76	76
*% survivors correctly predicted*	73	73
*% Error rate*	25.5	25.5

***Model 2 :* Predictors - Tumor Stage & Bcl-2; Variable Selection Technique—Iterative Stepwise Discriminant Analysis**		
**Combination of Variables**^f^ (Tumor stageb & Bcl-2 expression^d^)	**PHREG^e^**	**LDA^e^**
I & II + Bcl-2 negative	0.68	0.71
III & IV + Bcl-2 negative	0.24	0.26
I & II + Bcl-2 positive	0.77	0.82
III & IV + Bcl-2 positive	0.37	0.41
*% deaths correctly predicted*	76	76
*% survivors correctly predicted*	73	73
*% Error rate*	25.5	25.5

**Model 3 : Predictors - Bcl-2 & Tumor grad; Variable Selection Technique—PHREG Backward Selection & Iterative Stepwise Discriminant Analysis**		
**Combination of Variables**^g^ (Tumor grade^c^ & Bcl-2 expression^d^)	**PHREG^e^**	**LDA^e^**
Low + Bcl-2 negative	0.49	0.56
Low + Bcl-2 positive	0.67	0.74
High + Bcl-2 negative	0.24	0.15
High + Bcl-2 positive	0.44	0.22
*% deaths correctly predicted*	79	29
*% survivors correctly predicted*	53	87
**%*Error rate***	34	42

^a^ Variables were selected by the backward selection procedure in Proc PHREG in SAS.

^b^ Tumor stage was dichotomized as node negative (Stage I + II) and node positive (Stage III + IV).

^c^ Tumor differentiation dichotomized as low grade and high grade.

^d^ Phenotypic expression of Bcl-2 dichotomized as negative and positive as described in the *methods section*

^e^ Predicted outcome of survival probability beyond 5 years after surgery for the combination of variables.

^f^ Variables were selected using iterative bootstrapping sampling method described in the *methods section.*

^g^ Two variable selection procedures were tried (iterative bootstrapping and the backward selection) and the tumor stage was excluded from both the models.

**Table 2.** Significant Variables Obtained in a Proportional Hazards Regression Model.

**PHREG model including tumor stage as a variable**			**PHREG model excluding tumor stage as a variable**		
**Variables^a^**	**HR (95% CI)**	***P*value**	**Variables^a^**	**HR (95% CI)**	***P*value**
Tumor stage^b^	3.60 (2.58 – 5.027)	<0.0001			
Bcl-2 expression^c^	0.67 (0.493 – 0.92)	<0.0138	Bcl-2 expression^c^	0.58 (0.42 – 0.79)	0.0006
Tumor differentiation^d^	1.63 (1.11 – 2.38)	0.0112	Tumor differentiation^d^	2.03 (1.39 – 2.95)	0.0002

HR: hazard ratio; CI: confidence interval

^a^ Variables were selected using the stepwise discriminant analysis as described in the *methods section.*

^b^ Stage dichotomized as node negative (Stage I +II) and node positive (Stage III + IV).

^c^ Phenotypic expression of Bcl-2 dichotomized as negative and positive.

^d^ Tumor differentiation dichotomized as low grade and high grade.
